# Aortic Endovascular Stent IIIb Endoleak Combined With Sealed Aneurysm Rupture Mimicking a Paravertebral Retroperitoneal Tumor

**DOI:** 10.7759/cureus.16463

**Published:** 2021-07-18

**Authors:** Kyriakos Papavasiliou, Kyriakos Stavridis, Michael E Potoupnis, Ioannis Sarris, Eleftherios Tsiridis

**Affiliations:** 1 Orthopaedics, Aristotle University of Thessaloniki Medical School/ Papageorgiou General Hospital, Thessaloniki, GRC; 2 Vascular Surgery, Aristotle University of Thessaloniki/ Papageorgiou General Hospital, Thessaloniki, GRC

**Keywords:** abdominal aortic aneurysm, sealed rupture, iiib endoleak, endovascular stent, retroperitoneal tumor

## Abstract

The aim of this paper is to present the unique case of a sealed ruptured abdominal aortic aneurysm (AAA) with simultaneous type IIIb endoleak of an endovascular stent, mimicking a paravertebral retroperitoneal tumor. A 75-year-old male was referred to the tumor service of our tertiary orthopaedic department suffering from intractable low back pain with an onset of six months. He had undergone repair of an infrarenal AAA with an endovascular stent 5 years ago. Imaging studies depicted a large retroperitoneal mass adjacent to L2 and L3 vertebrae. The stent’s metal cage had no signs of wear or migration. The pathology report of a CT-guided core-needle biopsy he had undergone before his referral raised suspicion about possible AAA rupture. CT-angiography confirmed the existence of a ruptured AAA, accompanied by retroperitoneal blood loss and a (possibly) failed stent. The IIIb eroded stent was openly removed and a Y-type allograft was used to repair the defect. The patient reported immediate relief and was uneventfully discharged. He demised 4 years later due to reasons unrelated to the hereby reported condition. Increased awareness is warranted when dealing with anterior lumbar spine or retroperitoneal lesions. A close cooperation between a vascular and an orthopaedic/spine surgeon must always be sought.

## Introduction

Rupture of an Abdominal Aortic Aneurysm (AAA) is an uncommon cause of lower back pain. It is associated with extremely high mortality rate, unless patients receive immediate and highly specialized care. In the unusual case of chronic leakage, AAAs can become self-contained or “sealed”, leading to the development of chronic paravertebral hematomas, which in most cases are either asymptomatic or accompanied by minor symptoms [1]. Infrequently, an AAA rupture can occur following endovascular repair, leading to serious threat to the patient’s life [2].

We present the rare case of a sealed ruptured AAA with simultaneous type IIIb endoleak of an endovascular stent. To the best of our knowledge, this is a unique combination of a “double” lumen failure, clinically presented as a paravertebral retroperitoneal tumor.

## Case presentation

A 75-year-old Caucasian male patient was referred to the regional musculoskeletal tumor service of our tertiary hospital, suffering from intractable low back pain, with an onset of six months. He was diagnosed with a retroperitoneal tumor of unknown pathology, and had already undergone a CT-guided core-needle biopsy 10 days before his referral.

He had a significant past medical history (heavy smoking, hypertension, complete thyroidectomy for a benign tumor) and had undergone repair of an infrarenal AAA with an endovascular polyester/metal supported type Talent™ stent (World Medical, Medtronic Vascular, Sunrise-FLA, USA) 5 years ago. He was capable of full weight bearing, albeit with an antalgic gait and a scoliotic posture with the concave deformity towards his left side. No neurological deficit could be established and no signs of inflammation or infection were found. Active and passive flexion-extension movements of the lumbar spine were painful and he was unable to perform lateral bending or rotation. There was tenderness on palpation on the L1-L3 lumbar spinous processes. Abdominal examination revealed no pathological signs. The peripheral vascular supply was normal with bilaterally palpable popliteal, posterior tibialis and dorsalis pedis arteries. The venous drainage was also satisfactory. Routine blood tests were within normal range. A recently made Technetium99 bone scan, showed no uptake in any other part of the skeleton apart from the L2 and L3 vertebrae.

**Figure 1:**
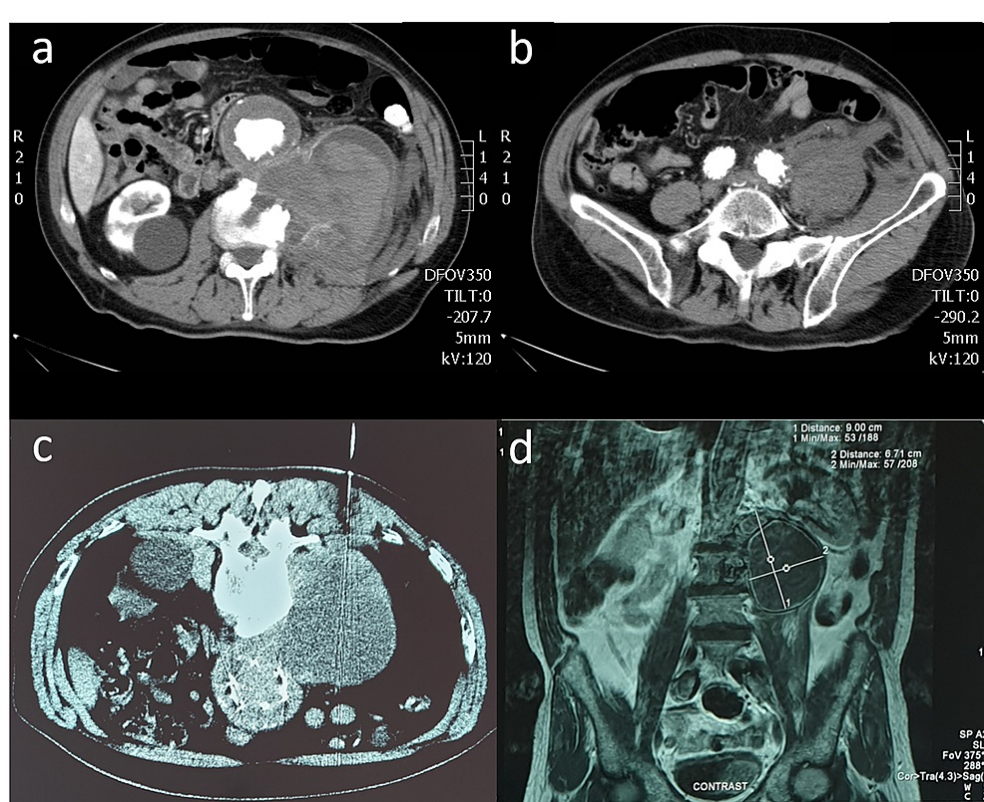
Pre-operative imaging workout of the patient (CT-scan, CT-guided core needle biopsy and MRI-scan) a & b: CT-scans, contrast enhanced, axial views. Notice the eroded body of the L2 vertebra and the large retroperitoneal mass being completely separated by the aneurysmatic sac (a) and the communication between the aneurysmatic sac of the left iliac branch junction of the stent and the retroperitoneal tumor at the L5-S1 level, depicting the rupture of the aneurysmatic sac (b). c: Computed tomography guided core-needle biopsy of the mass. Based on the biopsy track, the sample taken originated from the upper part of the mass, hence the aneurysmatic sac was not compromised at that level d: MRI-scan, contrast enhanced T2 weighted image, coronal view, depicting the large retroperitoneal mass being in direct contact with the L2 and L3 vertebrae that have signs of erosion.

Plain antero-posterior and lateral radiographs of the thoracic and lumbar spine revealed degenerative changes compatible with the patients΄age. Severe morphological changes of the anterior column of the L2 and L3 vertebrae were also evident, depicting bone erosion and signs of bone reformation. The metal cage of the stent had no signs of macroscopic wear or migration. The review of his (previously made) CT- and MRI-scans confirmed the existence of a large retroperitoneal mass in direct contact with the L2 and L3 vertebrae and the left iliopsoas muscle, due to which he was referred to our tumor service (Figure 1).

The lesion had distinct margins at its upper and mid part. At its lower part, however, there seemed to exist a communication between the lesion and the lower part of the abdominal aorta/left iliac artery. The lesion’s density was similar to that of soft tissue containing hemorrhagic material at different stages of the thrombosis procedure. The anterior parts of the L2 and L3 vertebral bodies also appeared significantly eroded and as if in a bone remodeling phase, thus confirming the findings seen on plain radiographs.

**Figure 2:**
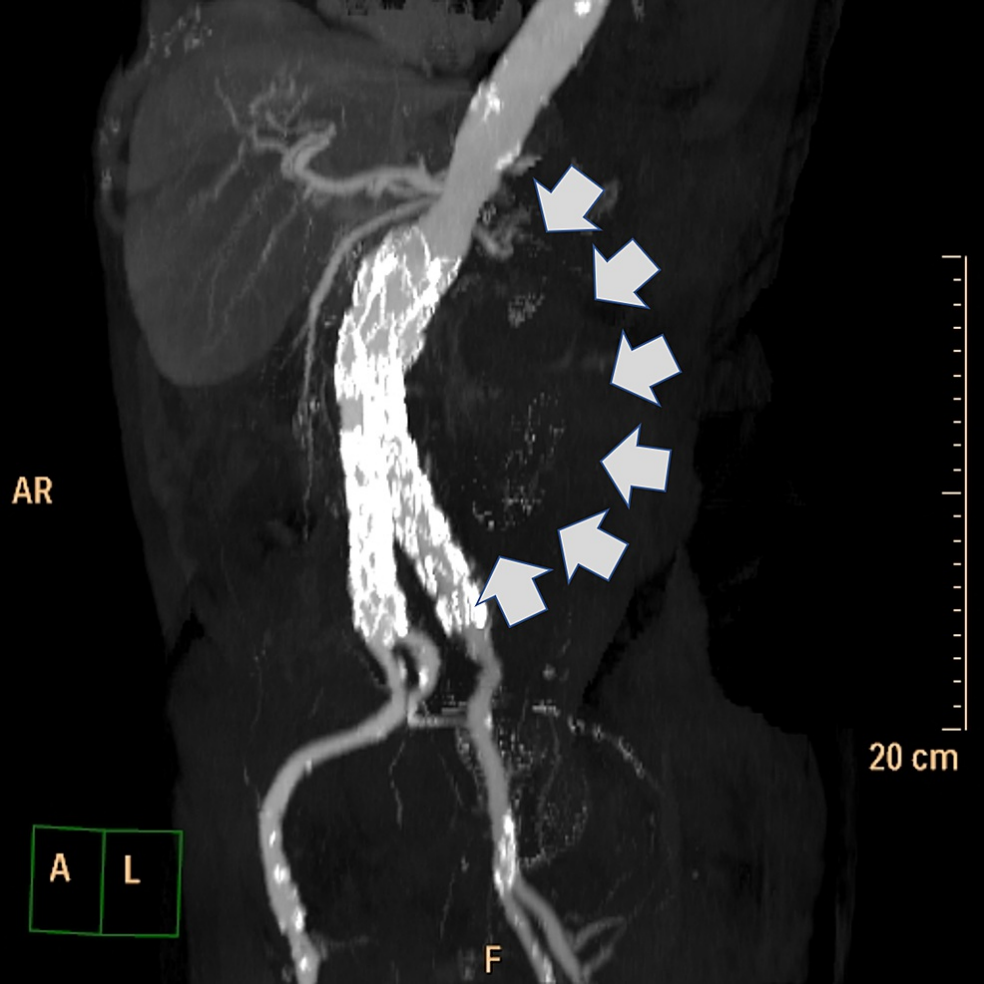
Pre-operative CT angiography of the patient. Computed tomography angiography (3D reconstruction) depicting the escape of the contrast media from the left iliac branch of the endovascular stent to the retroperitoneal mass under investigation (grey arrows).

The pathology report of the CT-guided core-needle biopsy was negative for malignancy or infection, describing nonetheless, the existence of inflammatory infiltrations by lymphocytes and neutrophils and a few foreign-body type multi-nucleated giant cells. The latter raised suspicion about a possible rupture of the AAA. A CT-angiography was performed next, confirming the existence of a ruptured AAA accompanied by retroperitoneal blood loss and (possibly) a failed stent (Figure 2).

**Figure 3:**
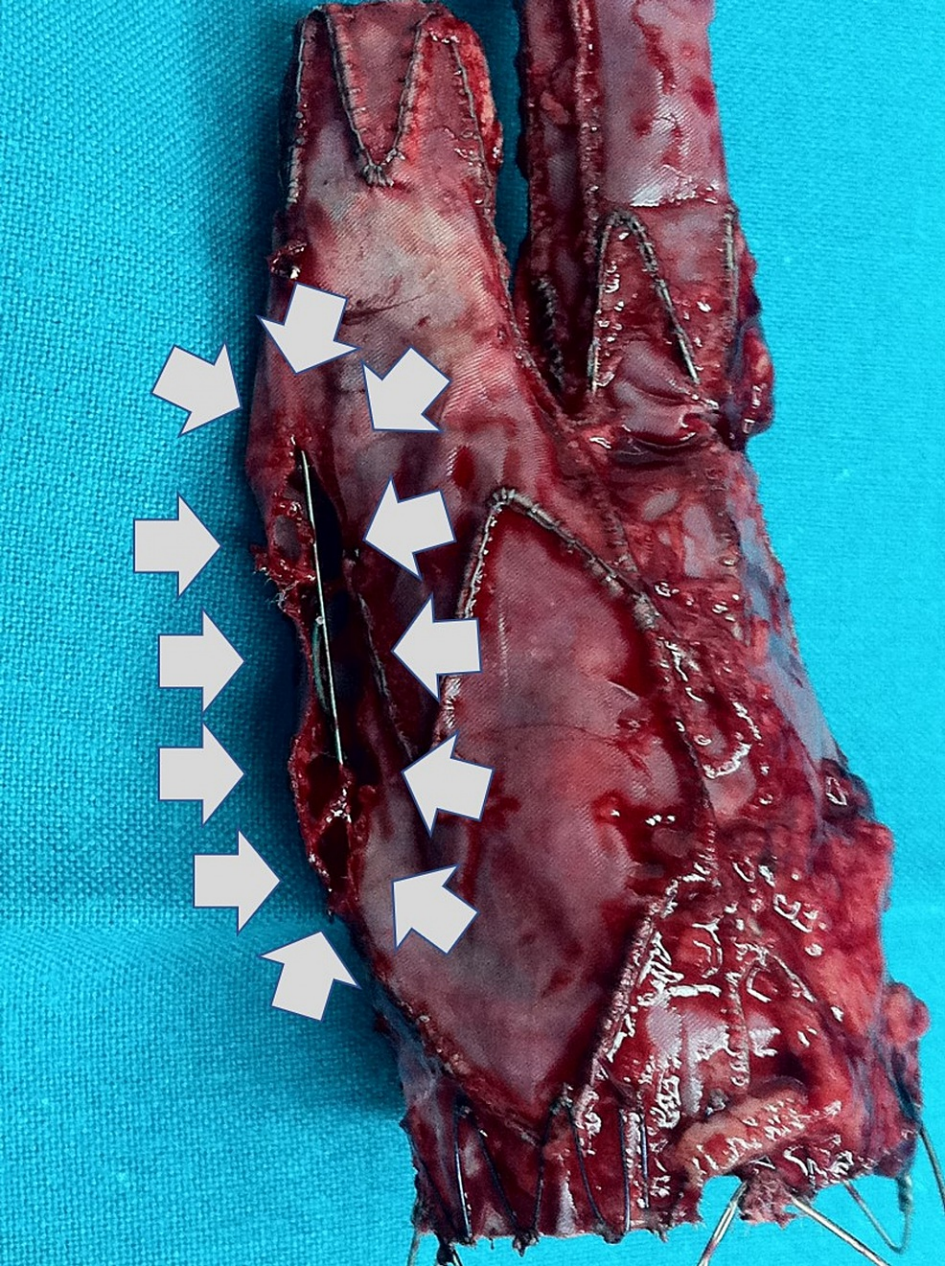
The retrieved failed endovascular stent. The retrieved endovascular stent, with an apparent longitudinal tear along its left iliac branch junction (grey arrows), inevitably leading to a type IIIb endoleak.

The patient was operated on as an emergency. Through a supra-infra umbilical abdominal transperitoneal approach, a large hematoma was identified and evacuated. A pulsating aneurysmal sac with a longitudinal tear of 1 cm at the level of the left iliac artery was found. The endovascular stent, which appeared eroded and with a wide longitudinal IIIb split at its aortic-iliac left junction (Figure 3), was removed and a Y-type Intervascular GORE® 18-9 stent (W.L. Gore and Associates Inc., Flagstaff, AZ, USA) was used to repair the defect. The allograft was anastomosed to the infrarenal aorta proximally and to the common femoral arteries distally (due to excessive atherosclerosis of the common iliac arteries).

The patient was transferred to the intensive care unit postoperatively, where he remained for 7 days. He developed acute renal failure, from which he recovered completely and he was uneventfully discharged two weeks postoperatively. On his follow-up visits, he occasionally reported minor lumbar pain, easily controlled by mild oral analgesics. He demised 4 years later by reasons unrelated to the hereby reported medical condition.

## Discussion

A paravertebral mass may pose several diagnostic and therapeutic dilemmas. Differential diagnosis may include tumors, potentially necessitating biopsy, and infection which may be caused by several pathogens [3]. In the latter case, symptoms and signs may not always be present or may be vague and/or misleading. In the majority of patients, the combination of CT- and MRI-scans can establish a diagnosis [4]. However, the differential diagnosis involves pathology originating from the abdominal aorta and other retroperitoneal anatomical structures. Orthopaedic/spinal surgeons frequently need to consult with vascular surgeons in order to exclude aortic pathologies such as pseudoaneurysms or self-contained ruptured AAAs before proceeding with elective spinal surgery [5].

Endovascular repair of AAAs has revolutionized their treatment. First described by Parodi et al. in 1991 [6], it is now considered a well-established and widely used treatment modality. This method appears to have satisfactory results mostly in the perioperative and mid-term period, based on the fact that it is a valuable alternative to open surgery with obviously much less morbidity. Its success rate is reported to be above 90%, with 30-day morbidity rates ranging from 1-9% [7]. Being the most common complication (10-50%) [7], endoleak can occur due to improper surgical technique and stent-grafts failure [8]. Rupture occurs in 1-1.5% of all previously treated by endovascular repair AAAs per year, usually with a fatal outcome [7]. Complication rates tend to increase significantly after the fourth postoperative year [9]. Close monitoring of patients who underwent endovascular repair facilitates the early identification of complications related to acute ruptures and enables their proper management.

The IIIb failure of a stent with a subsequently ruptured and contained aneurysm is rare and, to the best of our knowledge, has not been reported in literature. It is highly plausible that the endovascular stent failed first. Due to its IIIb endoleak, the aneurysmatic sac expanded and eventually ruptured. This inevitably contributed to the creation of a gradually expanding hematoma, which eventually got contained by the adjacent retroperitoneal tissues. This whole procedure led to the creation of a mass mimicking a paraspinal lesion. Τhe proximity of all surrounding soft-tissues and of the vertebral column and the rigidity of the latter must have probably further added to the “sealing” of the ruptured aneurysm. This assumption is further enhanced by the fact that signs of bone remodeling in the L2 and L3 vertebrae depicting a chronic condition, were evident in both the MRI- and the Technetium99 bone scans. The bone remodeling was probably caused by the continuously pulsating aneurysmatic sac (as confirmed intraoperatively) which was lying adjacent to the vertebral column, leading to the patient’s back pain. Last, but not least, it should be noted that the CT-guided biopsy, without prior consultation with a vascular surgeon, could have had extremely serious consequences for the patient, leading to a potentially fatal full rupture of the aneurysmal sac.

## Conclusions

Our rare case increases awareness when dealing with anterior lumbar spine/retroperitoneal tumors or tumorlike lesions. The proximity of this anatomic area to major vascular structures, necessitates meticulous pre-operative planning and consultation with several specialties, in order to avoid pitfalls with potentially catastrophic consequences.

Especially, when major vessels are involved in the pre-operative planning of orthopaedic operations and/or a patient had previously undergone a major vascular operation, a close co-operation between a vascular and an orthopaedic/spine surgeon must always be sought.
